# The Association between Insomnia and Insomnia Treatment Side Effects on Health Status, Work Productivity, and Healthcare Resource Use

**DOI:** 10.1371/journal.pone.0137117

**Published:** 2015-10-01

**Authors:** Marco DiBonaventura, Lance Richard, Maya Kumar, Anna Forsythe, Natalia M. Flores, Margaret Moline

**Affiliations:** 1 Kantar Health, New York, New York, United States of America; 2 Eisai Europe Ltd, Hatfield, United Kingdom; 3 Eisai Inc., Woodcliff Lake, New Jersey, United States of America; Peking University, CHINA

## Abstract

The aims of this study were (1) to compare health outcomes (i.e., health-related quality of life [HRQoL], productivity at work, and healthcare resource use visits) between those with insomnia and non-insomnia controls, (2) to compare health outcomes between those treated for insomnia and non-insomnia controls, and (3) to assess the prevalence of side effects of insomnia medications and their relationship with health outcomes. Data from the 2013 US (N = 75,000) and 5EU (N = 62,000) National Health and Wellness Survey (NHWS) were used. The NHWS is a patient-reported survey administered to a demographically representative sample of adults. Those who met DSM-V criteria for insomnia and, separately, those treated for insomnia were compared with equivalently sized control groups who were identified using a propensity score matching method. Outcomes included HRQoL (Short Form 36v2), productivity at work (Work Productivity and Activity Impairment—General Health questionnaire), and healthcare resource use visits in the past 6 months and were analyzed using one-way ANOVAs. Among those with treated insomnia, those with and without side effects were compared on health outcomes using general linear models controlling for confounding variables. Patients with insomnia (n = 4147) and treated insomnia (n = 2860) in the 5EU reported significantly worse HRQoL than controls (health utilities: 0.60 vs. 0.74; 0.60 vs. 0.74, respectively), greater overall work impairment (38.74% vs. 14.86%; 39.50% vs. 15.66%), and more physician visits in the past 6 months (9.10 vs. 4.08; 9.58 vs. 4.11). Similar findings were observed in the US. Among those treated for insomnia, 13.56% and 24.55% in the US and 5EU, respectively, were non-adherent due to side effects. In the US, non-adherence was associated with significantly worse HRQoL (health utilities: 0.60 vs. 0.64, p <.05) and greater overall work impairment (37.71% vs. 29.08%, p <.05), among other significant differences. These relationships were not significant in the 5EU. A significant burden of insomnia was observed in both the US and 5EU, and the association remained even after treatment. Non-adherence due to side effects was common and, in the case of the US, associated with significantly poorer health outcomes.

## Introduction

Prior research has suggested that approximately 30% of adults report difficulty falling asleep, difficulty maintaining sleep, or non-restorative sleep [1,2]. Several studies have suggested that patients who experience insomnia and symptoms of insomnia report a number of significant impairments. For instance, a study by Bolge and colleagues reported that respondents who had been diagnosed with insomnia and experienced their symptoms at least a few times per month reported significantly worse health-related quality of life (HRQoL) and greater impairment in work and leisure activities [3]. Moreover, a 2012 systematic review found a consistent negative effect of insomnia symptoms on HRQoL [4]. The significant effect of insomnia on individuals’ increased use of healthcare services and products and functional impairments has been shown to lead to a large societal economic burden [5].

There are a number of available medications for insomnia which have, in clinical studies, demonstrated significant improvements in sleep latency, total sleep duration, sleep quality, daytime functioning, and physical well-being [6–8]. However, these medications can be associated with both cognitive and psychomotor adverse events such as memory loss or loss of balance [8–12]. It remains unclear the extent to which the overall health of patients is restored (relative to those without insomnia) upon being treated for insomnia. Thus, it is critical to examine differences in HRQoL, work impairment, activity impairment, and healthcare resource use between those being treated for insomnia and those without insomnia.

Furthermore, given the added cognitive and physical burden of many available medications to treat insomnia [13–14], adverse events may have a direct effect on the health of the patients, undoing some of the very benefits the medications have on the sleep experience. However, studies have yet to examine the potential burden that adverse events may have on patient outcomes.

The aims of this study were threefold. The first aim was to examine the association between insomnia and health outcomes (i.e., HRQoL, productivity at work, and healthcare resource use) in the United States (US) and Western Europe, using the new criteria of the Diagnostic and Statistical Manual of Mental Disorders, Fifth Edition (DSM-V) to identify cases. Although this association has been examined previously, prior studies have used older DSM criteria (e.g., DSM-IV) to define insomnia. The second aim was to assess the residual effects of insomnia even after treatment. In other words, because insomnia treatment should improve the outcomes of patients to be, ideally, comparable with those without insomnia, we sought to quantify the extent of the remaining gap in health outcomes between treated patients and those without insomnia. The final aim was to explore the frequency of non-adherence due to adverse events (which can occur with current medications) and the extent to which these adverse events are associated with poorer health outcomes.

## Materials and Methods

### Data source

This study examines data from the 2013 National Health and Wellness Survey (NHWS). The NHWS is an annually conducted, Internet-based health questionnaire administered to a nationwide sample of adults (aged 18 or older) in the US and Western Europe (5EU; France, Germany, Italy, Spain, and UK) among other countries. The study protocol for both surveys was approved by an Institutional Review Board (Essex Institutional Review Board, Lebanon, NJ; Approval #: KH-NHWS-US13 and KH-NHWS-EU13). All respondents provided their consent to participate electronically.

In the US (N = 75,000), potential respondents for the NHWS are identified through the general panel of Lightspeed Research, a company that maintains various online respondent panels. All adults in the US aged 18 and over are eligible to join this panel and provided informed consent prior to answering any survey questions [15]. A stratified random sampling framework was used to ensure the final NHWS sample matched the demographic proportion of the US [16]. Several peer-reviewed publications have previously compared the NHWS with other governmental sources [3,17,18].

In the 5EU (N = 62,000), the method is largely the same. Within each country, the age and sex distributions are identified from the International Database of the US Census and mimicked during recruitment to ensure the demographic characteristics of the final NHWS sample are aligned with the population. Aside from minor country-specific differences (e.g., insurance types, income currencies), the survey in the 5EU and US are identical in the information captured as it pertains to this study.

### Sample

All respondents to the 2013 US (N = 75,000) and 5EU (N = 62,000) NHWS were included in the preliminary analyses. Measures from these surveys utilized in this study are detailed below.

### Primary measures

#### Insomnia

If respondents reported a diagnosis of insomnia and the use of a prescription medication, they were considered to have insomnia regardless of their symptom experience (because it is possible the treatment was successful at reducing the impact and frequency of their symptoms). If they did not report a diagnosis, insomnia was defined using the DSM-V criteria (see Table 1). Specifically, to be classified as having insomnia, a respondent had to indicate sufficient duration (3 months or more), frequency (at least 4 times a week), and impact (reporting their condition as either “moderate” or “severe” impact) of either sleep onset symptoms (“difficulty falling asleep”), sleep maintenance symptoms (“waking during the night and not being able to get back to sleep”, “waking up several times during the night”, or “waking up too early (such as before the alarm clock)”), or non-restorative sleep symptoms (“poor quality of sleep”). Respondents were excluded if they reported a diagnosis of other sleep disorders (i.e., narcolepsy, sleep-disordered breathing, circadian rhythm sleep disorder, or parasomnia).

**Table 1:** Insomnia and control group definitions.

Insomnia group definition	Control group definition
Meet DSM-V criteria for insomnia disorder	NO report of any **sleep onset** *, **sleep maintenance** **, or **non-restorative sleep symptoms** ***
** Sleep onset symptoms** * for at least 3 months and 4+ times per week and reporting their condition has a “moderate” or “severe” impact OR	NO diagnosis of narcolepsy
** Sleep maintenance symptoms** ** for at least 3 months and 4+ times per week and reporting their condition has a “moderate” or “severe” impact OR	NO diagnosis of sleep-disordered breathing
** Non-restorative sleep** *** for at least 3 months and 4+ times per week and reporting their condition has a “moderate” or “severe” impact OR	NO diagnosis of circadian rhythm sleep disorder
Diagnosed with insomnia AND using a prescription medication	NO diagnosis of parasomnia
NO diagnosis of narcolepsy	NO diagnosis of restless legs syndrome
NO diagnosis of sleep-disordered breathing	
NO diagnosis of circadian rhythm sleep disorder	
NO diagnosis of parasomnia	
NO diagnosis of restless legs syndrome	

*Sleep onset symptoms is defined by “difficulty falling asleep”.

**Sleep maintenance symptoms were defined by “waking during the night and not being able to get back to sleep”, “waking up several times during the night”, “waking up too early (such as before the alarm clock)”.

***Non-restorative sleep is defined by “poor quality of sleep”.

Control respondents were identified as those without sleep onset, sleep maintenance, or non-restorative sleep symptoms (i.e., no insomnia symptoms) and those without the same list of exclusionary sleep disorders as the insomnia group (e.g., narcolepsy, etc) (see Table 1). Respondents who reported insomnia symptoms but not of a sufficient duration, frequency, or impact based on DSM-V criteria were excluded entirely from the analyses as they did not meet criteria for either the insomnia group or the control group.

It should be noted that frequency and duration of symptoms were assessed in the NHWS among only those who reported having insomnia (although not necessarily having been diagnosed with insomnia). As a result, prevalence information for the insomnia group should be treated with caution, as it is likely an underestimate of all people in NHWS who would meet criteria if they were asked all symptom information.

#### Diagnosis, treatment, and non-adherence due to tolerability

All respondents who reported experiencing insomnia were asked whether they had been formally diagnosed and whether they were currently using a prescription medication to treat their insomnia. Respondents who reported taking a prescription medication were asked their level of adherence using the eight-item version of the Morisky Medication Adherence Scale (MMAS–8), tailored for insomnia medications [19]. Among those taking an insomnia medication, those who answered “yes” to the following MMAS–8 item were considered to be non-adherent due to adverse events: “Do you ever stop taking your medication because you feel worse?”.

#### Demographics and health characteristics

Age, sex (male or female), marital status (married/living with partner or not-married), education (university degree vs. less than university degree), household income, smoking status (currently smoke, former smoker vs. never smoker), alcohol use (currently drink vs. do not currently drink), and exercise behavior (currently exercise vs. do not currently exercise) were assessed as covariates. Additionally, comorbidities were measured using the Charlson Comorbidity Index (CCI) [20].

#### HRQoL

HRQoL was assessed via the Medical Outcomes Study 36-Item Short Form Survey Instrument version 2 (SF-36v2) [21]. The SF-36v2 is a multipurpose, generic HRQoL instrument comprised of 36 questions. Two component summary scores are generated from these times: the physical component summary (PCS) and the mental component summary (MCS). Each summary score is calculated using a norm-based scoring algorithm which sets the population mean at 50 and the standard deviation at 10. Higher scores represent better health status. The items from the SF-36v2 are also used to derive a preference-based health utility index that can be used for health economic assessment [22]. Using the SF-6D classification system, the responses to the SF-36v2 items are then converted to a health utility score, which conceptually varies from 0 (a health state equivalent to death) to 1 (a health state equivalent to perfect health). In other words, the health utility score gives an indication of the overall health state of the individual which varies, in theory, from 0 to 1, with higher scores indicating better health. There is a known floor effect of the health utility score derived from the SF-36v2, and it is not possible to achieve a score of 0 [23].

Past research has suggested that differences of 5.0 points on the norm-based domain scores, 3.0 points on the norm-based component summary scores, and .03 points on health utilities represent clinically meaningful differences [21,24]. The analyses in the present study will focus on MCS, PCS, and health utilities as outcomes.

#### Work productivity

Work productivity was assessed using the Work Productivity and Activity Impairment-General Health (WPAI-GH) questionnaire, a 6-item instrument which measures absenteeism (the percentage of work time missed because of one’s health in the past 7 days), presenteeism (the percentage of impairment experienced while at work in the past 7 days because of one’s health), overall work productivity loss (an overall impairment estimate that is a combination of absenteeism and presenteeism), and activity impairment (the percentage of impairment in daily activities because of one’s health in the past 7 days) [25]. Only respondents who reported being full-time or part-time employed provided data for the absenteeism, presenteeism, and overall work impairment scores. All respondents provided data for activity impairment.

#### Healthcare resource use

Healthcare utilization was assessed using a few different items. Specifically, respondents reported the number of traditional healthcare provider visits, the number of emergency room (ER) visits (i.e., "How many times have you been to the ER for your own medical condition in the past six months?"), and the number of times hospitalized in the past six months (i.e., "How many times have you been hospitalized for your own medical condition in the past six months?").

### Statistical analyses

**Fig 1:**
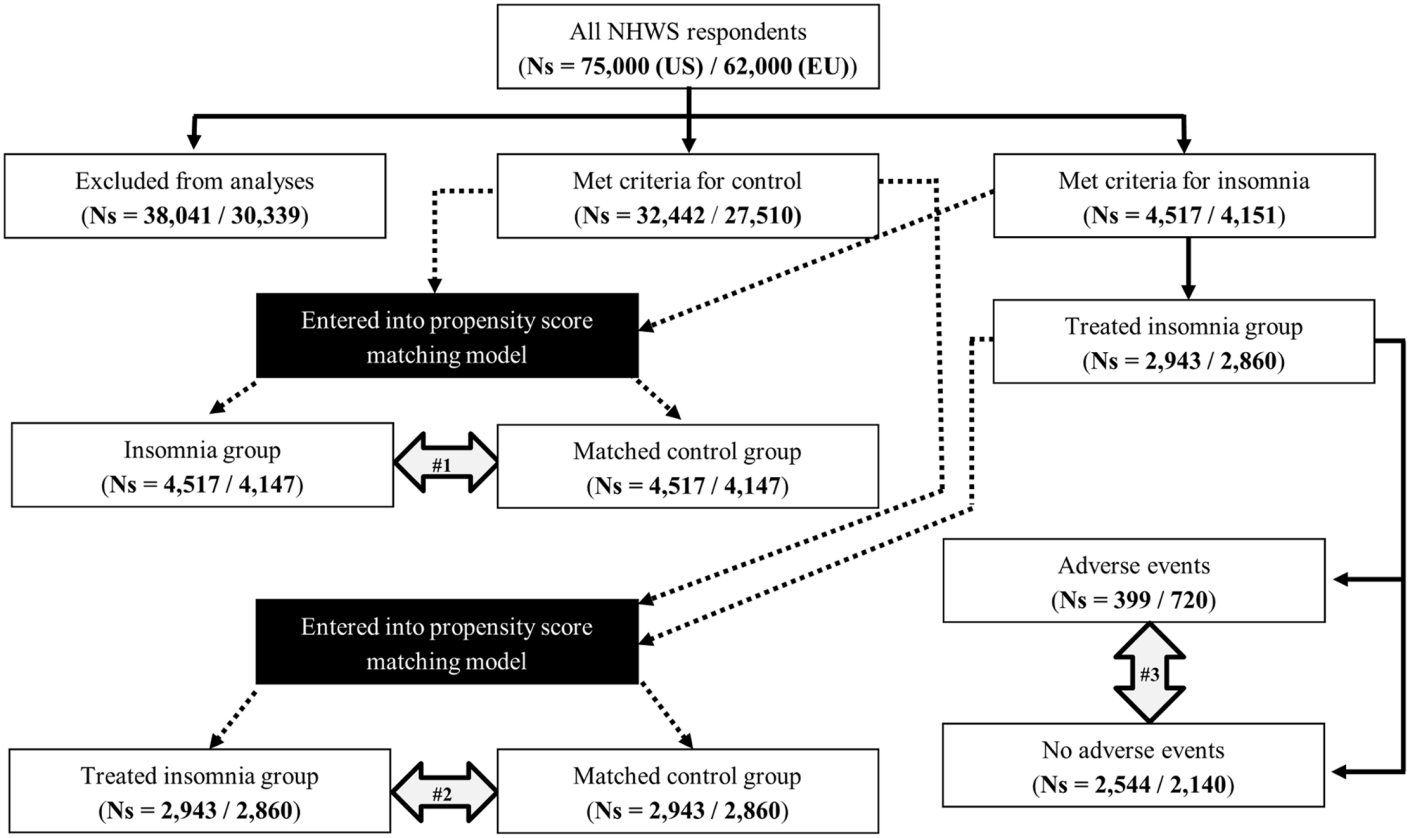
Analysis flowchart. Black solid arrows indicate groups which are direct subsets of other groups. Dotted arrows indicate which groups were entered into, and exited from, propensity score matching models. Grey double-arrows indicate the groups that were statistically compared in the main analyses.

Although the modeling approach is nearly identical, analyses were conducted separately in the US and 5EU. Any differences are noted explicitly in this section. Fig 1 graphically depicts the creation of all relevant subgroups and the overall analytical plan.

#### Association between insomnia and health outcomes: Analysis 1

Respondents who met criteria for insomnia (as described above and shown in Table 1) were compared with respondents who met criteria for the control group with respect to demographic and health history variables using chi-square tests and one-way analysis of variance (ANOVA) tests. Next, a 1:1 propensity score matching approach was used. Specifically, a logistic regression model was conducted predicting group membership (insomnia vs. control) from the following variables: age, sex, education, marital status, annual household income, employment status, smoking status, exercise behavior, alcohol use, and the CCI. Propensity score values were saved from this regression. Using a greedy matching algorithm, each case (i.e., patient with insomnia) was paired with a single control whose propensity score value is identical [26]. Cases and controls which were not matched were excluded from further analysis. Post-match, differences between the insomnia group and the matched control group were made with respect to all health outcomes (health status, work productivity, and healthcare resource use) using one-way ANOVA tests. This is illustrated as analysis #1 in Fig 1.

#### Residual effects of insomnia after treatment: Analysis 2

Respondents who met criteria for insomnia (as described above and shown in Table 1) and were treated with a prescription medication were compared with respondents who met criteria for the control group with respect to demographic and health history variables using chi-square tests and one-way ANOVA tests. Next, a 1:1 propensity score matching approach was used exactly as described above. Post-match, differences between the treated insomnia group and the matched control group were made with respect to all health outcomes (health status, work productivity, and healthcare resource use) using one-way ANOVA tests. This is illustrated as analysis #2 in Fig 1.

#### Prevalence of non-adherence due to adverse events and its association with health outcomes: Analysis 3

The prevalence of non-adherence due to adverse events was reported descriptively. For context, the prevalence of non-adherence reasons unrelated to adverse events was also reported. Among respondents with treated insomnia, those who reported and did not report non-adherence due to adverse events were compared with respect to demographic and health history variables using chi-square tests and one-way ANOVA tests. This is illustrated as analysis #3 in Fig 1. Differences between groups with respect to health outcomes were then analyzed within a regression framework (as the sample sizes of these subgroups were not conducive to a propensity score approach). Specifically, respondents who reported non-adherence due to an adverse event were compared with respondents who did not report non-adherence due to an adverse event on HRQoL variables using a general linear model, controlling for age, sex, smoking status, and the CCI. A similar comparison was made between groups on work productivity and healthcare resource use variables using generalized linear models, specifying for a negative binomial distribution and a log-link function (due to pronounced skew in these outcomes). As with the HRQoL models, these models controlled for age, smoking status, and the CCI. Adjusted means from all regression models were reported. All analyses were conducted in SASv9.3. Statistical significance for all tests was set at *p*<.05.

## Results

### Association between insomnia and health outcomes: Analysis 1

A total of 4,517 (6.0% of the US sample) met criteria for insomnia. Those with insomnia were significantly younger and more likely to be female, be unmarried, have less than a four-year education, have a lower household income, be unemployed, currently smoke, currently drink, and not exercise compared with those without insomnia (N = 32,442) (all *p*<.05; see Table 2). Respondents with insomnia also had a significantly greater comorbidity burden (*p*<.05).

**Table 2:** Demographics and health behaviors among respondents in the control group and respondents with insomnia.

	US			5EU		
Demographics	Control group (N = 32442)	Insomnia group (N = 4517)	P Value	Control group (N = 27510)	Insomnia group (N = 4151)	P Value
Female			*<*.*001*			*<*.*001*
Male (%)	17477 (53.9%)	1729 (38.3%)		14443 (52.5%)	1385 (33.4%)	
Female (%)	14965 (46.1%)	2788 (61.7%)		13067 (47.5%)	2766 (66.6%)	
Age			*<*.*001*			*<*.*001*
Mean ± SD	48.87 ± 16.92	47.93 ± 15.38		46.99 ± 16.28	48.96 ± 15.0	
Marital Status			*<*.*001*			*<*.*001*
Not married (%)	14889 (45.9%)	2542 (56.3%)		13515 (49.1%)	2342 (56.4%)	
Currently married (%)	17553 (54.1%)	1975 (43.7%)		13995 (50.9%)	1809 (43.6%)	
Education			*<*.*001*			*0*.*017*
Less than 4 Year University (%)	17666 (54.5%)	2976 (65.9%)		20045 (72.9%)	2951 (71.1%)	
4 Year University or Higher (%)	14776 (45.5%)	1541 (34.1%)		7465 (27.1%)	1200 (28.9%)	
Income			*<*.*001*			
<$25K (%)	5451 (16.8%)	1190 (26.3%)		--	--	*--*
$25K to <$50K (%)	8060 (24.8%)	1285 (28.4%)		--	--	*--*
$50K to <$75K (%)	6497 (20.0%)	844 (18.7%)		--	--	*--*
$75K or more (%)	9678 (29.8%)	944 (20.9%)		--	--	*--*
Decline to answer (%)	2756 (8.5%)	254 (5.6%)		--	--	*--*
Income						*<*.*001*
Income below the median (%)	--	--	*--*	11678 (42.5%)	2269 (54.7%)	
Income above the median (%)	--	--	*--*	11522 (41.9%)	1385 (33.4%)	
Decline to answer (%)	--	--	*--*	4310 (15.7%)	497 (12.0%)	
Employment Status			*<*.*001*			*<*.*001*
Not Employed (%)	14216 (43.8%)	2534 (56.1%)		11277 (41.0%)	2241 (54.0%)	
Employed (%)	18226 (56.2%)	1983 (43.9%)		16233 (59.0%)	1910 (46.0%)	
Smoking Status			*<*.*001*			*<*.*001*
Current smoker (%)	4610 (14.2%)	1242 (27.5%)		6205 (22.6%)	1412 (34.0%)	
Former Smoker (%)	8738 (26.9%)	1424 (31.5%)		8018 (29.1%)	1277 (30.8%)	
Never Smoker (%)	19094 (58.9%)	1851 (41.0%)		13287 (48.3%)	1462 (35.2%)	
Alcohol consumption			*<*.*001*			*0*.*043*
Currently drink (%)	20461 (63.1%)	3019 (66.8%)		20843 (75.8%)	3085 (74.3%)	
Do not currently drink (%)	11981 (36.9%)	1498 (33.2%)		6667 (24.2%)	1066 (25.7%)	
Exercise behavior			*<*.*001*			*<*.*001*
Don't currently exercise (%)	10138 (31.2%)	1811 (40.1%)		10652 (38.7%)	1996 (48.1%)	
Currently exercise (%)	22304 (68.8%)	2706 (59.9%)		16858 (61.3%)	2155 (51.9%)	
Charlson Comorbidity Index			*<*.*001*			*<*.*001*
Mean ± SD	0.30 ± 0.82	0.67 ± 1.24		0.21 ± 0.63	0.49 ± 1.02	

The results in the 5EU were largely the same as the US. A total of 4,151 (6.7% of the 5EU sample) met criteria for insomnia. Those with insomnia were significantly older and more likely to be female, be unmarried, have a lower household income, be unemployed, currently smoke, and not exercise than controls (N = 27,510) (all *p*<.05; see Table 2). Respondents with insomnia also had a significantly greater comorbidity burden (*p*<.05).

After conducting a propensity score matching process, all health outcome differences between respondents with insomnia and matched controls were significant in each geography (all *p*<.05; see Table 3). Differences in health status were of sufficient magnitude to be considered clinically significant (i.e., 3 and 0.03 point differences between groups in MCS/PCS and health utility scores, respectively). Work and activity-related impairment was more than double for those with insomnia compared with matched controls. In the US, healthcare resource use was between 50% (0.39 vs. 0.22 ER visits in the past 6 months) and 120% (6.70 vs. 3.05 physician visits in the past 6 months) higher for those with insomnia. In the 5EU, all forms of resource use were more than twice as high for those with insomnia compared to matched controls.

**Table 3:** Health outcome differences between those with insomnia and matched controls.

	US			5EU		
Health Outcome	Matched control group (N = 4517)	Insomnia group (N = 4517)	P Value	Matched control group (N = 4147)	Insomnia Group (N = 4147)	P Value
ER visits	0.22 ± 1.71	0.39 ± 1.10	*<*.*001*	0.16 ± 0.75	0.39 ± 1.62	*<*.*001*
Hospital visits	0.12 ± 0.70	0.20 ± 0.76	*<*.*001*	0.11 ± 0.55	0.27 ± 1.41	*<*.*001*
Physician visits	3.05 ± 4.52	6.70 ± 8.69	*<*.*001*	4.08 ± 5.62	9.10 ± 9.84	*<*.*001*
Absenteeism %	3.28 ± 12.94	8.17 ± 20.67	*<*.*001*	4.38 ± 16.76	12.31 ± 27.42	*<*.*001*
Presenteeism %	10.74 ± 21.07	27.96 ± 27.20	*<*.*001*	12.11 ± 21.65	32.13 ± 27.91	*<*.*001*
Overall work impairment %	12.85 ± 24.10	32.18 ± 30.86	*<*.*001*	14.86 ± 25.88	38.74 ± 32.98	*<*.*001*
Activity impairment	19.34 ± 27.27	42.92 ± 31.39	*<*.*001*	20.85 ± 26.96	46.85 ± 30.85	*<*.*001*
MCS (SF-36v2)	51.34 ± 9.33	40.20 ± 12.66	*<*.*001*	49.47 ± 9.10	36.45 ± 11.75	*<*.*001*
PCS (SF-36v2)	50.74 ± 9.24	45.37 ± 11.07	*<*.*001*	51.54 ± 8.76	46.04 ± 10.67	*<*.*001*
Health utilities (SF-36v2)	0.76 ± 0.13	0.62 ± 0.12	*<*.*001*	0.74 ± 0.13	0.60 ± 0.11	*<*.*001*

ER = Emergency room; MCS = Mental component summary; PCS = Physical component summary.

### Residual effects of insomnia after treatment: Analysis 2

In the US, a total of 2,943 met criteria for both having insomnia and being treated (65.2% of those with insomnia). Compared with those without insomnia (N = 32,442), those with treated insomnia were significantly more likely to be female, be unmarried, have less than a four-year education, have a lower household income, be unemployed, currently smoke, currently drink, and not exercise (all *p*<.05). Respondents with treated insomnia also had a significantly greater comorbidity burden (*p*<.05). No differences were observed with respect to age (49.11 years vs. 48.87 years for those treated with insomnia and controls, respectively, p = .46).

The results in the 5EU were largely similar; 68.9% of those who met criteria for insomnia were treated (N = 2,860). Compared with those without insomnia (N = 27,510), those with treated insomnia were significantly older and more likely to be female, be unmarried, have a lower household income, be unemployed, currently smoke, and not exercise (all *p*<.05). Respondents with treated insomnia also had a significantly greater comorbidity burden (*p*<.05).

In both the US and 5EU, all differences between respondents with treated insomnia and matched controls were significant (all *p*<.05; see Table 4). Differences with respect to HRQoL all exceeded established cutoffs for clinical significance. In particular, MCS levels were more than a full standard deviation lower among those with treated insomnia. Work and activity-related impairment was between twice and three-times as high for those with treated insomnia relative to matched controls. Similarly, healthcare resource use was more than twice as high for those with treated insomnia compared to matched controls.

**Table 4:** Health outcome differences between those with treated insomnia and matched controls.

	US			5EU		
Health Outcome	Matched control group (N = 2943)	Treated insomnia (N = 2943)	P Value	Matched control group (N = 2860)	Treated insomnia (N = 2860)	P Value
ER visits	0.18 ± 1.00	0.42 ± 1.16	*<*.*001*	0.16 ± 0.73	0.44 ± 1.81	*<*.*001*
Hospital visits	0.12 ± 0.80	0.24 ± 0.86	*<*.*001*	0.12 ± 0.72	0.30 ± 1.63	*<*.*001*
Physician visits	3.26 ± 4.34	7.60 ± 9.29	*<*.*001*	4.11 ± 5.58	9.58 ± 9.83	*<*.*001*
Absenteeism %	2.34 ± 11.18	8.57 ± 20.81	*<*.*001*	4.83 ± 18.29	12.79 ± 27.63	*<*.*001*
Presenteeism %	10.41 ± 20.12	26.92 ± 27.45	*<*.*001*	12.21 ± 21.73	32.71 ± 28.21	*<*.*001*
Overall work impairment %	11.78 ± 22.41	31.39 ± 31.15	*<*.*001*	15.66 ± 26.76	39.50 ± 33.21	*<*.*001*
Activity impairment	19.45 ± 27.15	42.03 ± 31.72	*<*.*001*	21.66 ± 27.43	46.03 ± 30.69	*<*.*001*
MCS (SF-36v2)	52.16 ± 9.23	41.59 ± 12.43	*<*.*001*	49.56 ± 9.27	37.06 ± 11.70	*<*.*001*
PCS (SF-36v2)	50.29 ± 9.49	45.30 ± 10.84	*<*.*001*	51.14 ± 8.85	46.20 ± 10.25	*<*.*001*
Health utilities (SF-36v2)	0.76 ± 0.13	0.63 ± 0.13	*<*.*001*	0.74 ± 0.13	0.60 ± 0.11	*<*.*001*

ER = Emergency room; MCS = Mental component summary; PCS = Physical component summary.

### Prevalence of non-adherence due to adverse events and its association with health outcomes: Analysis 3

Among those with insomnia using a prescription medication, 399 (13.6%) reported being non-adherent due to adverse events in the US (39.7% reported non-adherence due to forgetfulness and/or inconvenience, the other forms of non-adherence assessed by the MMAS–8). These patients were younger, more likely to currently smoke, and had a significantly lower comorbidity burden than patients who did not report being non-adherent due to adverse events (all *p*<.05). No other differences were observed. The frequency of non-adherence due to adverse events was higher in the 5EU, with 24.5% (N = 702) reporting (contrasted with 37.0% who reported non-adherence due to forgetfulness and/or inconvenience). As with the US, these patients were significantly younger and had a lower comorbidity burden (all *p*<.05). However, these patients were also more likely to be female, have less than a university degree, be employed, currently drink, and exercise (all *p*<.05).

In the US, after adjusting for confounding variables, no differences between groups were observed with respect to healthcare resource use (despite trends for increasing healthcare resource use). However, patients who reported non-adherence due to adverse events reported significantly more presenteeism, overall work impairment, activity impairment, and also reported significantly worse MCS, PCS, and health utilities (all *p*<.05; see Table 5).

**Table 5:** Adjusted health outcome differences between those with and without adverse events among those with insomnia taking a medication in the US.

Health Outcome	No adverse events (N = 2544)	Adverse events (N = 399)	P Value
ER visits	0.36	0.44	*0*.*164*
Hospital visits	0.20	0.26	*0*.*149*
Physician visits	7.39	8.08	*0*.*168*
Absenteeism	7.92	9.30	*0*.*421*
Presenteeism	24.78	33.12	*0*.*001*
Overall work impairment	29.08	37.71	*0*.*002*
Activity impairment	40.85	46.53	*0*.*002*
MCS	42.00	38.98	*<*.*001*
PCS	45.46	44.31	*0*.*042*
Health utilities	0.64	0.60	*<*.*001*

ER = Emergency room; MCS = Mental component summary; PCS = Physical component summary.

All models controlled for age, sex, smoking status and CCI.

However, in the 5EU, few differences were observed between those who reported being non-adherent due to adverse events and those who did not prior to covariate adjustment. Those who reported non-adherence due to side effects reported fewer physician visits and greater PCS scores. After covariate adjustment, the number of physician visits and activity impairment were slightly lower while PCS scores were slightly higher, but no other differences were observed.

## Discussion

One of the aims of the study was to re-examine the association between insomnia and health outcomes in the US and 5EU, using the new criteria of the DSM-V. The results suggest a significant humanistic and economic burden within both regions, reflecting findings from past research [3,4]. Although adjustments were made for economic and health behavioral factors as part of the analyses, it is worth emphasizing the profile of patients with insomnia. Respondents with insomnia were of lower socioeconomic standing and exhibited poorer health behaviors. The causal relationship among these factors (i.e., does insomnia contribute to poorer lifestyle choices or the reverse?) was beyond the scope of the current study. Nevertheless, the findings suggest a significant degree of complexity when attempting to properly manage these patients as there may be a number of additional environmental and behavior factors which could limit the improvement of treatment interventions.

Respondents with insomnia reported lower physical and mental HRQoL to a clinically significant degree relative to controls matched on demographic and health history variables. The association between insomnia and HRQoL was particularly strong, with differences between groups approaching a full standard deviation, in some cases. Indeed, these HRQoL effects were larger than what has been reported in the literature for other sleep disorders. The MCS and PCS values in our study (40 and 45 in the US) were lower than those reported for severe sleep apnea (44 and 48, respectively) [27]. Also, compared with a study by Moline and colleagues [28], which also used the NHWS, insomnia had a more detrimental effect on HRQoL than any of the following individual sleep symptoms: middle-of-the-night awakening, difficulty falling asleep, non-restorative sleep, waking up several times, and waking up too early (which were up to 5 MCS and 2 PCS points lower than controls rather than 11 and 5 points lower, respectively, in our study) [28].

Similarly, respondents with insomnia reported significantly more absenteeism, presenteeism, overall work impairment, activity impairment, and healthcare resource use in the past six months. Differences between groups were large, with respondents with insomnia often reporting more than double the impairment and healthcare resource use relative to matched controls. These effects were much larger than individual sleep symptoms observed in the literature [28]. However, these work-related effects were not as strong as those observed among patients with narcolepsy [29].

The ER visit/hospitalization differences are particularly noteworthy as insomnia would likely not be directly associated with an increase in these events, yet, rates were significantly higher among those with insomnia than matched controls. Although the reasons for ER visits and hospitalizations were not captured in NHWS, it could be hypothesized that patients with insomnia reported a greater number of clinical encounters due to accidents, which may be related to either the symptoms of insomnia [30] and/or the side effects of certain insomnia medications [8–12]. Further research is necessary to explore this relationship. As with work-related effects, our study reported much stronger findings than the effect of individual sleep symptoms [31] but less dramatic effects when compared with narcolepsy [32].

An additional aim of this study was to understand the residual effect of insomnia among those who have been treated. In ideal circumstances, proper treatment would allow the patient with insomnia to have health outcomes comparable to those without insomnia. However, a wide gap in health outcomes remained between patients with insomnia and using treatment and matched controls. As with analyses examining the overall burden of insomnia, respondents with insomnia who were treated reported significantly worse health status (also to a clinically relevant degree), greater work and activity impairment, and greater healthcare resource use. Naturally, there may be a selection bias partially influencing the results. Those who are currently being treated may have a greater severity of insomnia than those with insomnia who are not being treated (thus artificially exacerbating the differences between those with insomnia and controls). Moreover, the data were not longitudinal, so it is not possible to test the degree to which health outcomes improved for those who were being treated. These results do not necessarily suggest that treatments are not effective but, rather, suggest that their improvement is not necessarily restoring patient outcomes (particularly health status) to a level comparable to those without insomnia. Future research could explore a similar analysis, yet categorize patients into responders versus non-responders to examine how the patient outcomes may vary.

In part, this gap could be explained by adverse events of treatments, examination of which was the final objective of this study. Although available medications have demonstrated clinical efficacy, many come at a cost of additional cognitive and psychomotor effects [8–12]. The presence or absence of specific adverse events was not assessed directly in the NHWS, but a total of 13.6% in the US and 24.6% in 5EU reported not taking their insomnia medication due to adverse events. Because this measure assessed adverse events indirectly, it most likely reflects a conservative estimate of those affected by adverse events. For instance, it is highly likely that there are some respondents who experienced adverse events but continued taking their medication as prescribed. Such respondents would not be included in our group of non-adherent due to adverse events. It is also possible that some respondents stopped taking medication altogether due to adverse events and these respondents would have been excluded from the calculation (since only those currently treated were included). Regardless, these figures highlight the extent to which the profile of current insomnia treatments affects patient adherence.

Those who reported non-adherence due to adverse events reported significantly worse HRQoL and work productivity and more healthcare resource use compared to those who did not report non-adherence due to adverse events. Interestingly, the results suggest a difference between the US and 5EU. Although non-adherence due to adverse events was less common in the US, it was more detrimental to health outcomes than in the 5EU, where there were largely no differences.

However, it is difficult to make comparisons across the US and 5EU for several reasons. First, the available medications vary among regions (e.g., ramelteon, which has a tolerable profile, was approved in the US at the time of data collection but not in Europe). Additionally, even if the same medication is available in both regions, its use can vary substantially. For instance, research has suggested that zolpidem, eszopiclone, zaleplon (i.e., Z medications) are the most commonly prescribed insomnia medications in the US, however, their use is more restricted in Europe due to the potential for abuse [30–31]. Finally, there are differences with respect to which medications are available by a prescription and which are available over-the-counter in the 5EU, the latter being taken by the patient with less physician oversight. For all these reasons, it is difficult to directly compare regions with respect to the frequency and effect of adverse events. As complete information on the medication experience of patients was beyond the present study, future work would need to investigate the adverse event experience (and associated non-adherence) for specific medications across these regions to have a more accurate comparison.

### Limitations

The current study highlights the HRQoL, work productivity, and healthcare resource use effects on individuals with insomnia. Despite these strengths, there are limitations to the current study. All data were self-reported and no verification of an insomnia diagnosis, treatment usage, or healthcare resource use was available. Recall biases could have affected the measurement of these variables. The study was also cross-sectional, thus causality between insomnia, treatments, and health outcomes cannot be formally tested. For example, it is possible that experiencing work-related impairment for health reasons unrelated to insomnia may lead to insomnia symptoms because of the psychological effects (e.g., anxiety, depression) of not working to one’s ability or expectations. Similarly, repeated healthcare visits (again, unrelated to insomnia) may cause insomnia symptoms, mediated by psychological factors. Given the cross-sectional design, we are unable to tease apart the direction of these associations (or the degree to which one direction contributes vis-à-vis another). Other third variables not accounted for in the analysis could have also influenced the results. Finally, although the NHWS is demographically representative, it is unclear the extent to which this analytical sample generalizes to the various insomnia subpopulations in each country.

## Conclusions

In sum, a significant association was observed between the presence of insomnia and HRQoL, work productivity loss, and healthcare resource use in both the US and 5EU. Further, these associations remained even after treatment. This study also found that non-adherence due to adverse events was common and, in the case of the US, associated with significantly poorer patient outcomes.
